# Supplementary material for: Heterogeneous, population-level drug-tolerant persisters exhibit ion-channel remodeling and ferroptosis susceptibility
Source: bioRxiv. 2026 May 8:2022.02.03.479045. Preprint. [Version 3] doi: 10.1101/2022.02.03.479045 (PMC13105065; doi:10.1101/2022.02.03.479045)
Supplement: Supplement 1 — Figure S1: Gene ontology analysis of single-cell transcriptomic clusters. (A) Ten single-cell clusters identified by the ‘Seurat’ software package used in this work (see Materials and Methods). In this UMAP projection, the “untreated small” (UTS) cluster corresponds to Seurat cluster 8, the “untreated large” (UTL) cluster to Seurat clusters 1, 2, 4, and 9, the “idling small” (IS) cluster to Seurat cluster 6, and the “idling large” (IL) cluster to Seurat clusters 0, 3, 5, and 7. (B) GO analysis of differentially expressed genes between the UTS and UTL clusters in the UMAP single-cell transcriptomics space. (C) Same as B but applied to the IS and IL clusters. Figure S2: DNA barcode abundances and distributions across untreated and idling DTP states. (A) Heatmap of relative barcode abundances for all experimental replicates of the untreated and idling DTP populations. The heatmap is organized by decreasing barcode abundance in replicate 1 (R1) of the untreated condition. RPM: reads per million. (B) Number of unique barcodes in each treatment condition and number of shared barcodes between conditions. Lines correspond to the means of three experimental replicates. A minimum cutoff of 100 counts per million (CPM) was used. Note that the number of unique barcodes is higher in the drug-treated (idling) condition than in the untreated cells. We attribute this to biological noise, i.e., more lineages went extinct, by chance, in the untreated sample than in the idling sample. Overall, we interpret these data as evidence that lineages are not being clonally selected for upon drug treatment. (C) Proportional sharing of barcodes among experimental replicates for untreated and idling DTP populations. (D) Overlay of single cells from 20 barcoded lineages on the transcriptomic UMAP space from FIG. 1A. Colored contours (red and blue) reflect cell density for both treatment conditions. Figure S3: Quality control of bulk ATAC-seq data and correlation analyses of transcriptome- and epi [file media-1.pdf]

Figure S1

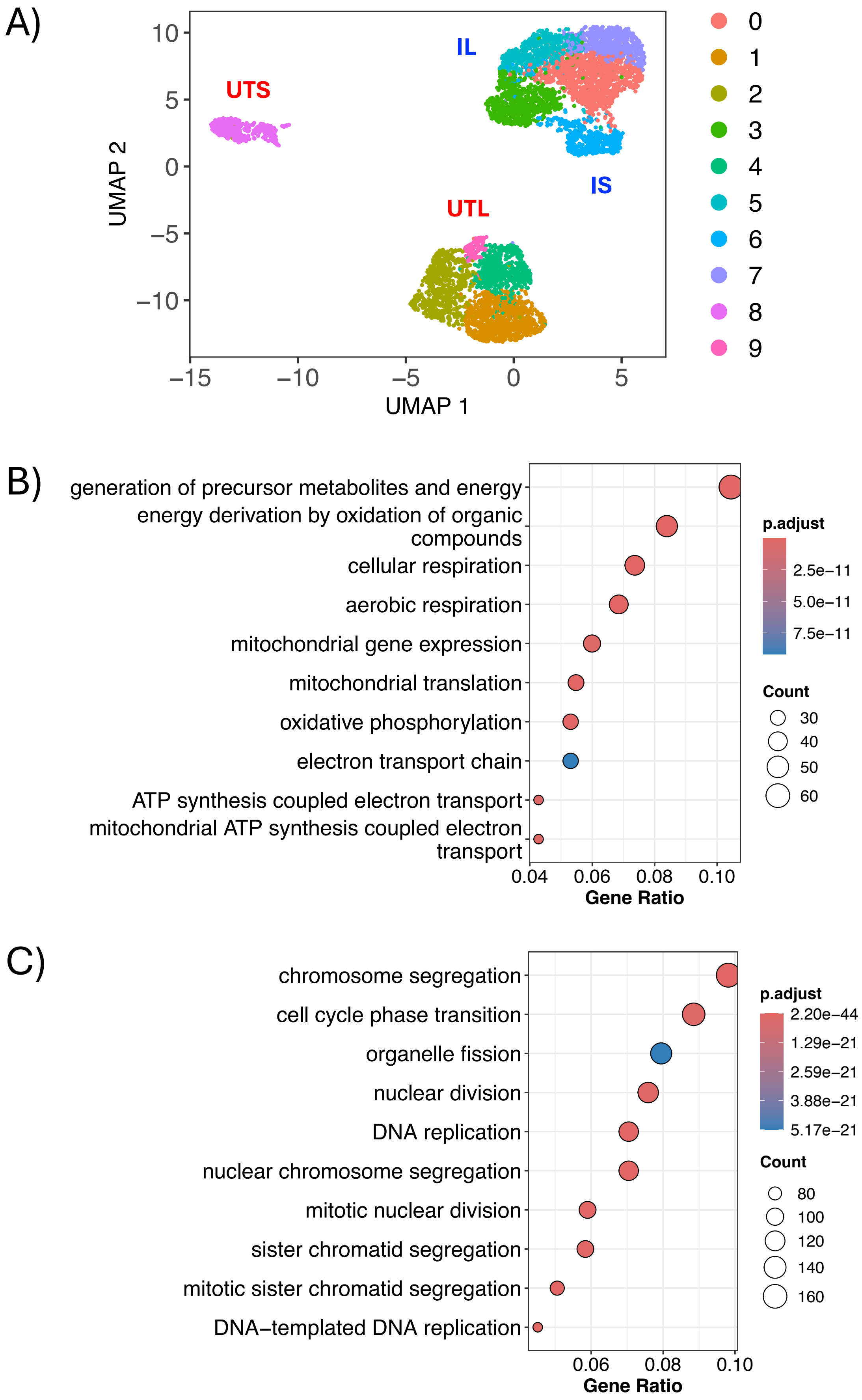
A)
UTS
IL
IS
UTL
UMAP 2
UMAP 1
10
5
0
−5
−10
−15
−10
−5
0
5
0
1
2
3
4
5
6
7
8
9
B)
generation of precursor metabolites and energy
energy derivation by oxidation of organic compounds
cellular respiration
aerobic respiration
mitochondrial gene expression
mitochondrial translation
oxidative phosphorylation
electron transport chain
ATP synthesis coupled electron transport
mitochondrial ATP synthesis coupled electron transport
0.04
0.06
0.08
0.10
Gene Ratio
p.adjust
2.5e−11
5.0e−11
7.5e−11
Count
30
40
50
60
C)
chromosome segregation
cell cycle phase transition
organelle fission
nuclear division
DNA replication
nuclear chromosome segregation
mitotic nuclear division
sister chromatid segregation
mitotic sister chromatid segregation
DNA−templated DNA replication
0.06
0.08
0.10
Gene Ratio
p.adjust
2.20e−44
1.29e−21
2.59e−21
3.88e−21
5.17e−21
Count
80
100
120
140
160

Figure S2

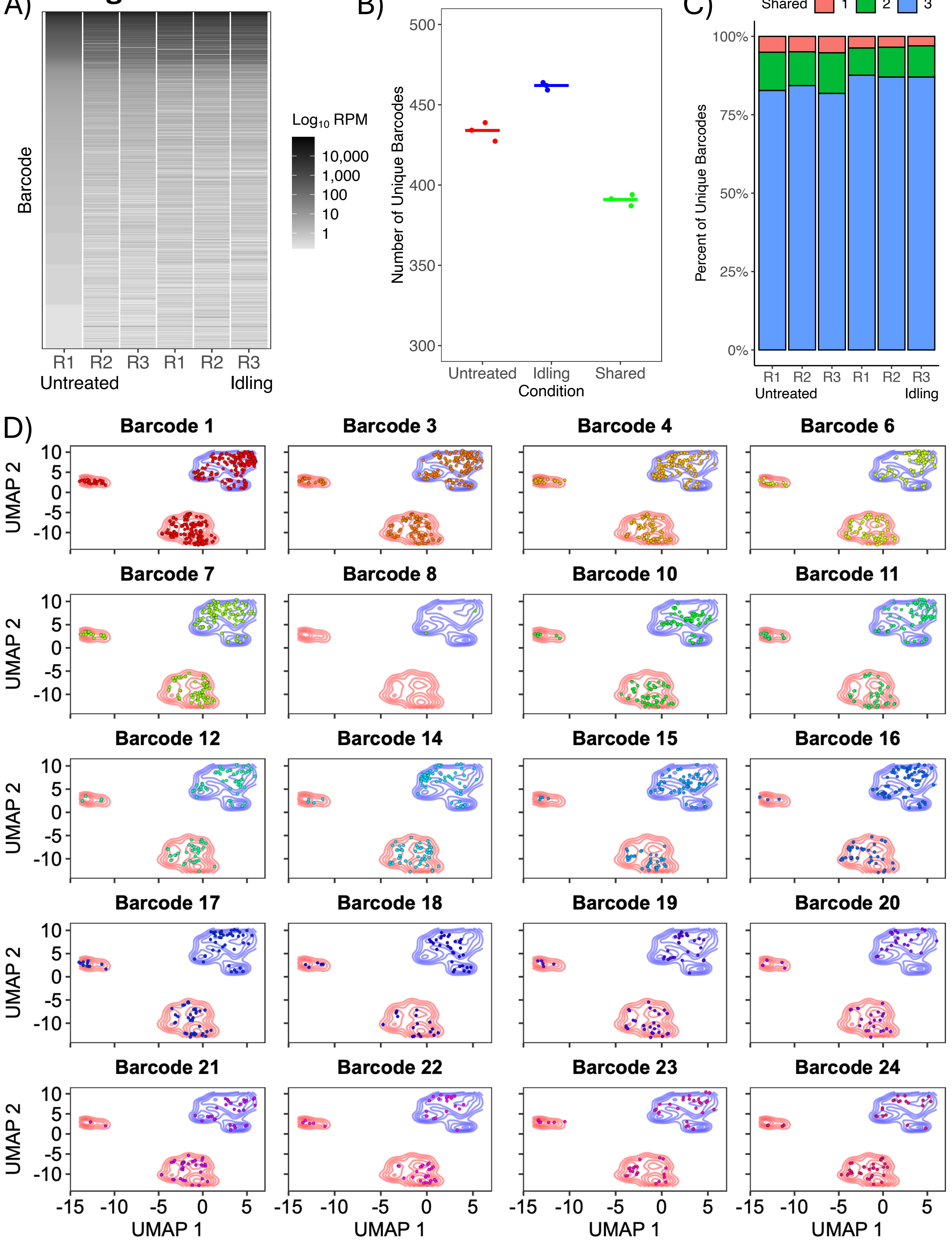

A)
Barcode
Log10 RPM
10,000
1,000
100
10
1
R1 R2 R3 R1 R2 R3
Untreated
Idling
B)
Number of Unique Barcodes
500
450
400
350
300
Untreated Idling Shared
Condition
C)
Shared 1 2 3
100%
75%
50%
25%
0%
Percent of Unique Barcodes
R1 R2 R3 R1 R2 R3
Untreated
Idling
D)
Barcode 1
Barcode 3
Barcode 4
Barcode 6
Barcode 7
Barcode 8
Barcode 10
Barcode 11
Barcode 12
Barcode 14
Barcode 15
Barcode 16
Barcode 17
Barcode 18
Barcode 19
Barcode 20
Barcode 21
Barcode 22
Barcode 23
Barcode 24
UMAP 2
UMAP 1
-15 -10 -5 0 5
10 5 0 -5 -10

Figure S3

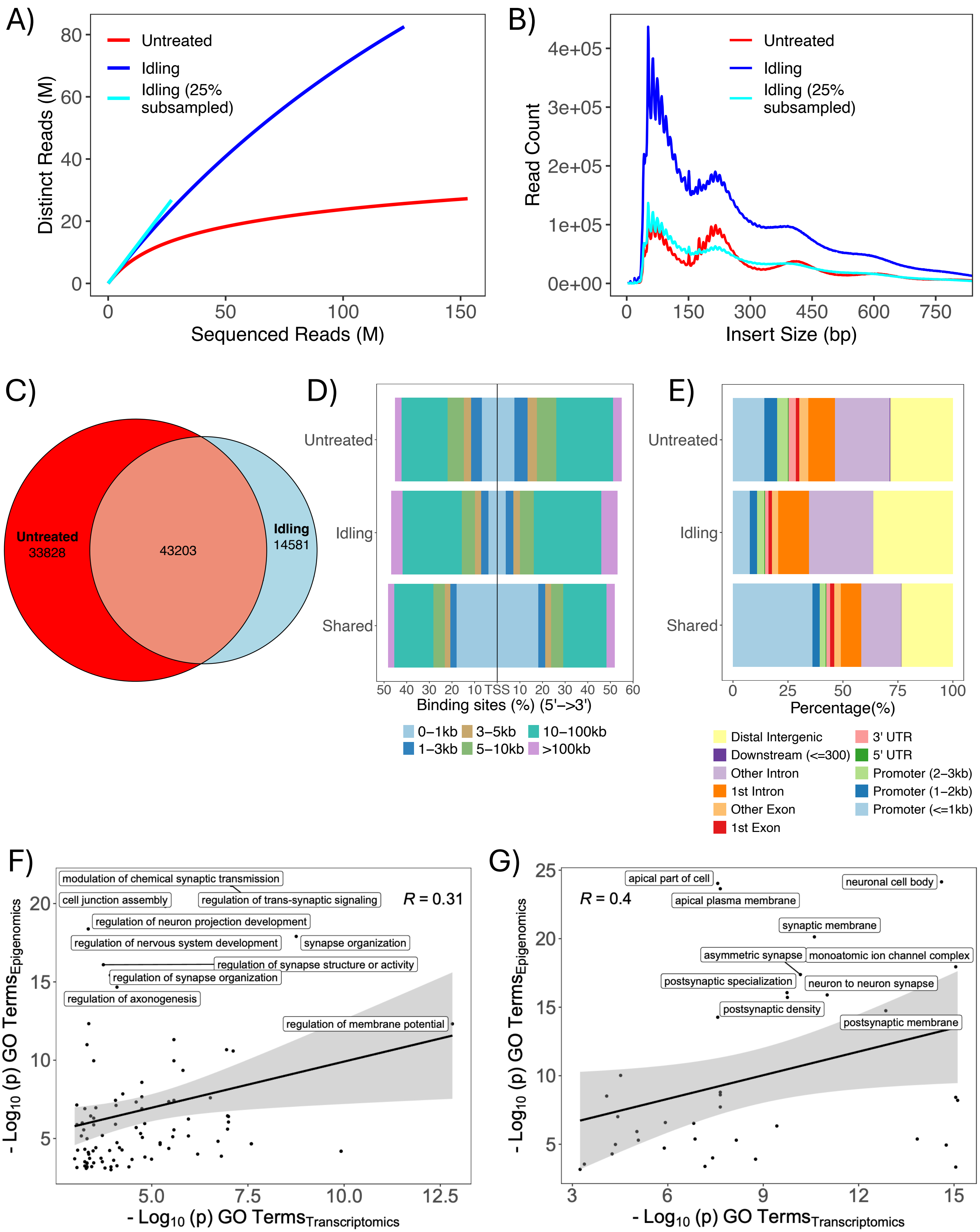

A)
Untreated
Idling
Idling (25% subsampled)
Distinct Reads (M)
Sequenced Reads (M)
B)
Read Count
Insert Size (bp)
C)
Untreated 33828
43203
Idling 14581
D)
Untreated
Idling
Shared
Binding sites (%) (5'->3')
0–1kb
1–3kb
3–5kb
5–10kb
10–100kb
>100kb
E)
Percentage(%)
Distal Intergenic
Downstream (<=300)
Other Intron
1st Intron
Other Exon
1st Exon
3' UTR
5' UTR
Promoter (2–3kb)
Promoter (1–2kb)
Promoter (<=1kb)
F)
R = 0.31
modulation of chemical synaptic transmission
cell junction assembly
regulation of trans-synaptic signaling
regulation of neuron projection development
regulation of nervous system development
synapse organization
regulation of synapse structure or activity
regulation of synapse organization
regulation of axonogenesis
regulation of membrane potential
- Log10 (p) GO Terms Epigenomics
- Log10 (p) GO Terms Transcriptomics
G)
R = 0.4
apical part of cell
apical plasma membrane
neuronal cell body
synaptic membrane
asymmetric synapse
monoatomic ion channel complex
postsynaptic specialization
neuron to neuron synapse
postsynaptic density
postsynaptic membrane
- Log10 (p) GO Terms Epigenomics
- Log10 (p) GO Terms Transcriptomics

Figure S4

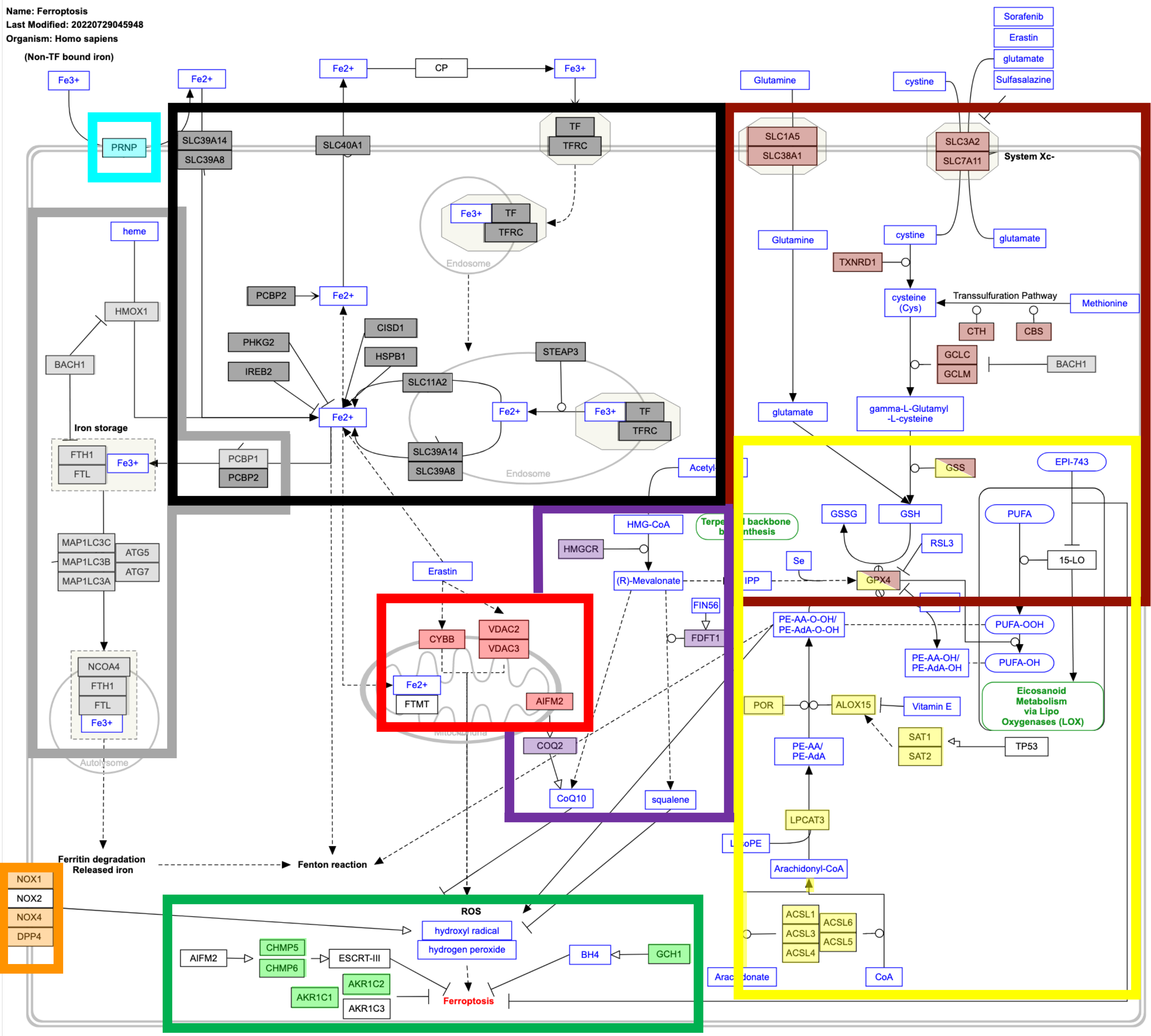

Gene ontology

- PUFA
- Terpenoid Biosynthesis
- Autolysosome Ferritin Storage
- Endosome Ferrous Transfer
- Mitochondria
- Superoxide Generation
- Inhibition of Ferroptosis
- Ferric acid reduction
- Glutathione Metabolism

wikipathways.org/pathways/WP4313.html
